# Differential Fungal Susceptibility of *Aspergillus oryzae* to Aomori Hiba Heartwood and Sapwood

**DOI:** 10.3390/s26041191

**Published:** 2026-02-12

**Authors:** Tsuyoshi Yoda

**Affiliations:** 1 Hirosaki Industrial Research Institute, Aomori Prefectural Industrial Technology Research Center, 1-1-8, Ougimachi, Hirosaki City 036-8104, Aomori, Japan; tsuyoshi_yoda@aomori-itc.or.jp; 2 Industrial Research Institute, Aomori Prefectural Industrial Technology Research Center, 221-10, Yamaguchi Nogi, Aomori City 030-0142, Aomori, Japan; 3 The United Graduate School of Agricultural Sciences, Iwate University, 3-18-8, Ueda, Morioka City 020-8550, Iwate, Japan

**Keywords:** image-based sensing, digital image analysis, fungal susceptibility, food safety, wood extractives, *Aspergillus oryzae*

## Abstract

**Highlights:**

**What are the main findings?**

- Aomori hiba heartwood shows significantly higher fungal susceptibility suppression against *Aspergillus oryzae* than sapwood.
- The difference in fungal susceptibility correlates quantitatively with higher Soxhlet-extractable content in heartwood.

**What are the implications of the main findings?**

- Wood-part-dependent extractive distribution is a key factor governing fungal susceptibility of traditional Japanese timber.
- The results support selective utilization of heartwood for hygienic and food-related wood applications.

**Abstract:**

The antifungal properties of wood are often attributed to extractives that differ between heartwood and sapwood; however, quantitative evaluation methods remain limited. In this study, we investigated differences in fungal susceptibility between heartwood and sapwood of Aomori Hiba (*Thujopsis dolabrata var. hondae*) using solvent extraction, fungal growth inhibition assays, and digital image analysis. Heartwood and sapwood were distinguished based on anatomical characteristics and color, and extractives were obtained using ethanol as the solvent. Antifungal activity was evaluated against *Aspergillus oryzae* by monitoring fungal growth on culture media. Quantitative image analysis was applied to grayscale images to assess fungal growth inhibition, enabling objective comparison between samples. The results demonstrated that heartwood extracts consistently exhibited stronger fungal growth inhibition than sapwood extracts, which correlated with higher extractive contents. Image-derived metrics effectively captured differences in fungal growth that were not readily discernible by visual inspection alone. These findings demonstrate that digital image analysis can be effectively integrated with fungal susceptibility assays and extractive measurements to provide a practical framework for preliminary screening of antifungal potential in wood-derived materials.

## 1. Introduction

Wood-derived natural materials have garnered increasing attention as sustainable antimicrobial resources for food, packaging, and hygiene applications, driven by concerns over synthetic preservatives and their environmental impact. Among Japanese coniferous species, Aomori hiba (*Thujopsis dolabrata var. hondae*) is well known for its exceptional resistance to decay and insects, characteristics that have historically supported its use in culturally important buildings such as Buddhist temples and Shinto shrines [1]. These properties are generally attributed to the accumulation of bioactive extractives in heartwood, which play a key role in wood durability and biological resistance, as discussed in classical studies on wood extractives and heartwood formation [2,3]. In the case of Aomori hiba, specific bioactive extractives, including hinokitiol and related tropolone compounds, have been identified as major contributors to its broad antimicrobial activity [1,4,5,6,7]. More broadly, numerous natural products and wood-derived compounds have been reported to exhibit antimicrobial activity, with some studies suggesting enhanced effects through synergistic or potentiation mechanisms [6,7,8]. Although the antimicrobial properties of Aomori hiba have been widely recognized, quantitative comparisons of functional differences between heartwood and sapwood remain limited.

In wood science, the distinction between heartwood and sapwood is fundamental. Heartwood formation is accompanied by the accumulation of extractive components, whereas sapwood primarily serves physiological transport functions [9,10]. These extractive components in heartwood have been proposed to play an active defensive role against biological deterioration, rather than being merely metabolic by-products [9]. Differences in extractive composition and concentration are closely linked to natural durability and resistance to biological deterioration [9,10,11,12]. Accordingly, extractable components are widely recognized as key contributors to the chemical and biological properties of wood materials [13]. Wood extractives are known to exhibit diverse biological activities, including antimicrobial and antifungal effects, as summarized in previous studies, including reports in the Japanese literature [14]. Numerous studies have reported antimicrobial or fungal susceptibility of wood extractives; however, many focus on specific compounds or qualitative observations rather than direct quantitative comparisons between heartwood and sapwood [4,5,11]. For example, antimicrobial activity of heartwood extractives has been reported for other conifer species such as sugi (*Cryptomeria japonica*) against several fungi and bacteria [15].

Fungal deterioration of wood materials is of particular relevance in humid environments and food-related contexts. Resistance of wood-based materials to mold fungi has been investigated for several species, highlighting the importance of extractives in suppressing fungal growth [16]. Nevertheless, systematic evaluations targeting food-related filamentous fungi remain scarce. *Aspergillus oryzae* plays a central role in traditional Japanese fermentation processes and is therefore an important organism from both industrial and hygienic perspectives [17,18]. Despite its significance, differences in fungal susceptibility of heartwood versus sapwood against *A. oryzae* have not been quantitatively examined.

The objective of this study was to clarify whether heartwood and sapwood of Aomori hiba exhibit distinct antifungal activities against *A. oryzae* and to determine whether any observed differences correspond to differences in the amount of extractable components. Hinokitiol-related compounds (thujaplicins) have long been recognized as important bioactive extractives in Aomori hiba, and their analytical determination has been studied since the mid-20th century [19]. To address this, fungal susceptibility was evaluated using a standardized agar diffusion assay, and extractable components were quantified by Soxhlet extraction, a widely used method for assessing wood extractives [13]. By focusing on a direct and quantitative comparison between heartwood and sapwood, this study aims to provide fundamental data supporting the rational utilization of Aomori hiba resources.

In recent years, sensor-based approaches for evaluating biological and material-related properties have expanded beyond conventional electrochemical or spectroscopic devices to include optical and image-based sensing methodologies. In particular, digital image analysis has emerged as a practical sensing strategy that enables quantitative, observer-independent measurement of biological responses using widely accessible hardware, such as digital cameras and flatbed scanners [20,21]. In this context, image-derived parameters, including intensity profiles, area, and spatial distribution of biological features, can be regarded as sensing outputs analogous to signals obtained from traditional sensors.

Image-based sensing approaches have been increasingly applied in food science and hygiene-related research, where rapid screening and low-cost evaluation are often required. Examples include the assessment of microbial growth, biofilm formation, and surface contamination on food-contact materials [22,23]. Compared with chemical sensors that target specific analytes, image-based sensing provides an integrative response reflecting the overall biological effect of complex material compositions, making it particularly suitable for preliminary screening and comparative evaluation. Moreover, the use of digital image analysis minimizes subjective bias and enhances reproducibility, both of which are critical requirements for sensor-based measurement.

From this perspective, agar diffusion assays combined with quantitative image analysis can be interpreted not merely as biological tests, but as functional bio-sensing systems in which fungal growth inhibition serves as a measurable response signal [21]. When coupled with standardized image acquisition and analysis procedures, such systems enable consistent comparison of material-dependent biological effects. Importantly, this approach does not require specialized instrumentation, thereby supporting its applicability in practical settings related to food safety, storage environments, and hygienic material selection.

Despite the relevance of image-based sensing in these fields, its application to the quantitative evaluation of wood-derived antimicrobial properties, particularly with respect to heartwood–sapwood differences, remains limited [24]. Therefore, positioning antifungal evaluation within a sensing framework may provide new insights into the functional characterization of traditional wood materials and their potential hygienic applications.

## 2. Materials and Methods

### 2.1. Wood Samples

Aomori Hiba (*Thujopsis dolabrata var. hondae*) wood was obtained from locally sourced timber. Heartwood and sapwood were distinguished based on their anatomical position within the trunk and characteristic visual color differences, following established criteria for heartwood formation. Specifically, heartwood was identified as the inner, darker-colored region rich in extractives, whereas sapwood corresponded to the outer, lighter-colored region of the trunk, as described in previous anatomical studies [25,26].

**Figure 1:**
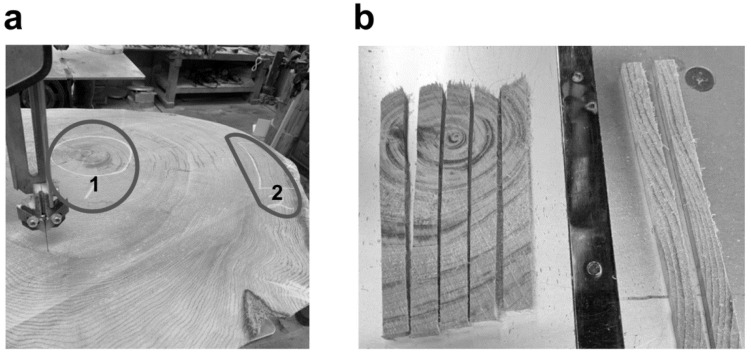
Macroscopic appearance and preparation of Aomori hiba samples used in this study. (**a**) Representative cross-sectional image of Aomori hiba showing the central part (heartwood; left, Part 1) and the outer part (sapwood; right, Part 2). (**b**) Preparation of wood samples for antifungal experiments: Part 1 (heartwood, left) and Part 2 (sapwood, right).

Wood samples were provided by a commercial supplier in the form of square pieces with dimensions of approximately 1 cm × 1 cm. The samples were air-dried and used as received without additional milling or sieving to maintain uniformity in sample size and shape across all experiments. The wood samples were prepared from timber pieces of different diameters as follows: (A) heartwood from a trunk with a diameter of approximately 60 cm, (B) sapwood from the same trunk, and (C) heartwood from a trunk with a diameter of approximately 30 cm. The cutting positions are illustrated in Figure 1.

### 2.2. Fungal Strain and Culture Conditions

*Aspergillus oryzae* was used as a representative food-related filamentous fungus. Spores were prepared from 1 g of commercial granular koji starter (used for Akita Konno miso production) by suspending the spores in 45 mL of distilled water. The spore suspension was further diluted tenfold with distilled water prior to inoculation. Potato dextrose agar (PDA) supplemented with chloramphenicol (50 ppm) was used as the culture medium to suppress bacterial growth.

### 2.3. Fungal Susceptibility Assay

Fungal susceptibility of heartwood and sapwood samples was evaluated using an agar diffusion assay. A volume of 0.7 mL of the diluted spore suspension was evenly spread onto the surface of PDA plates and allowed to air-dry. Wood samples (1 cm × 1 cm × 0.5 cm) were then placed individually or in combination on the inoculated agar surface. The plates were covered and incubated at 30 °C.

Three types of wood samples were examined: (A) heartwood from a trunk with a diameter of approximately 60 cm, (B) sapwood from the same trunk, and (C) heartwood from a trunk with a diameter of approximately 30 cm. After 3 days of incubation, fungal growth inhibition was evaluated by the formation of clear zones around the wood samples.

Images of the agar plates were acquired for quantitative analysis, and fungal susceptibility was quantified as the diameter of the inhibition (clear) zone formed around each sample. The image acquisition and analysis procedures were based on methods established in our previous studies involving camera-based image analysis and quantitative grayscale evaluation [27,28,29,30]. Inhibition zones were measured from captured images using ImageJ software (version 1.54f, National Institutes of Health, Bethesda, MD, USA). Images were acquired with the built-in camera of a smartphone (Xperia XZ, Sony Corporation, Tokyo, Japan) inside a clean bench under fluorescent lighting conditions. The same imaging setup and acquisition conditions were applied to all samples to ensure consistency. All experiments were performed in triplicate (*n* = 3).

### 2.4. Soxhlet Extraction and Quantification of Extractable Components

Extractable components were quantified by Soxhlet extraction following established procedures for wood extractives [13]. Dried wood samples were subjected to continuous extraction using diethyl ether as an organic solvent, which preferentially extracts low-molecular-weight, relatively non-polar wood extractives. After solvent removal, the mass of the extract was determined gravimetrically. Extractable component content was expressed as milligrams of extract per milligram of dry wood sample (mg mg^−1^), representing a normalized yield rather than an absolute mass.

For heartwood, extractable component contents were 4.89, 10.22, and 6.74 mg mg^−1^ (*n* = 3). For sapwood, corresponding values were 3.42, 3.93, and 3.37 mg mg^−1^ (*n* = 3).

### 2.5. Statistical Analysis

Data are presented as mean ± standard deviation (SD). Differences between heartwood and sapwood samples were evaluated using a two-tailed Student’s *t*-test. When variances were unequal, Welch’s correction was applied. A *p*-value of less than 0.05 was considered statistically significant. All statistical analyses were performed using R statistical software (version 4.1.3, R Foundation for Statistical Computing, Vienna, Austria; https://www.r-project.org/, accessed on 2 February 2026). Analyses were conducted within RStudio (version 2026.01.0, Posit Software, Boston, MA, USA). For measurements performed in triplicate (*n* = 3), standard deviation values were calculated and reported to illustrate experimental variability.

## 3. Results

### 3.1. Fungal Susceptibility Against Aspergillus oryzae

**Figure 2:**
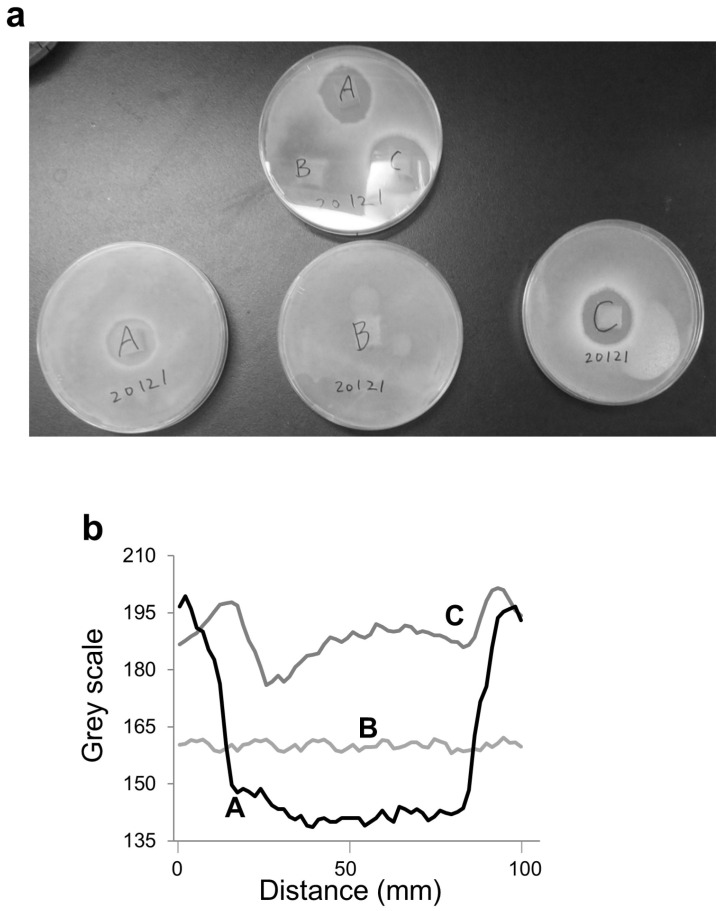
Part-dependent fungal susceptibility of Aomori hiba against *Aspergillus oryzae* (koji mold). (**a**) Representative images of inhibition (clear) zones formed around wood samples in the agar diffusion assay. (**b**) Representative grayscale intensity profiles of the clear zones obtained by linear grayscale analysis across the inhibition zones. Wood samples A (heartwood from a trunk with a diameter of approximately 60 cm) and C (heartwood from a trunk with a diameter of approximately 30 cm) were prepared from the central region of Aomori hiba, whereas sample B was prepared from the outer region (sapwood). Grayscale intensity was measured along a linear region crossing the center of each wood sample to visualize spatial changes associated with fungal growth inhibition.

Figure 1 shows the macroscopic appearance and preparation of heartwood and sapwood samples of Aomori hiba used in this study. Fungal susceptibility was evaluated using these samples under identical experimental conditions. As shown in Figure 2a, heartwood samples exhibited larger inhibition (clear) zones against *A. oryzae* than sapwood samples, indicating stronger fungal susceptibility. Representative grayscale intensity profiles obtained by image analysis further supported this visually observed difference between heartwood and sapwood (Figure 2b). Overall, fungal growth was more strongly suppressed in the presence of heartwood than sapwood.

### 3.2. Soxhlet-Extractable Component Content

**Figure 3:**
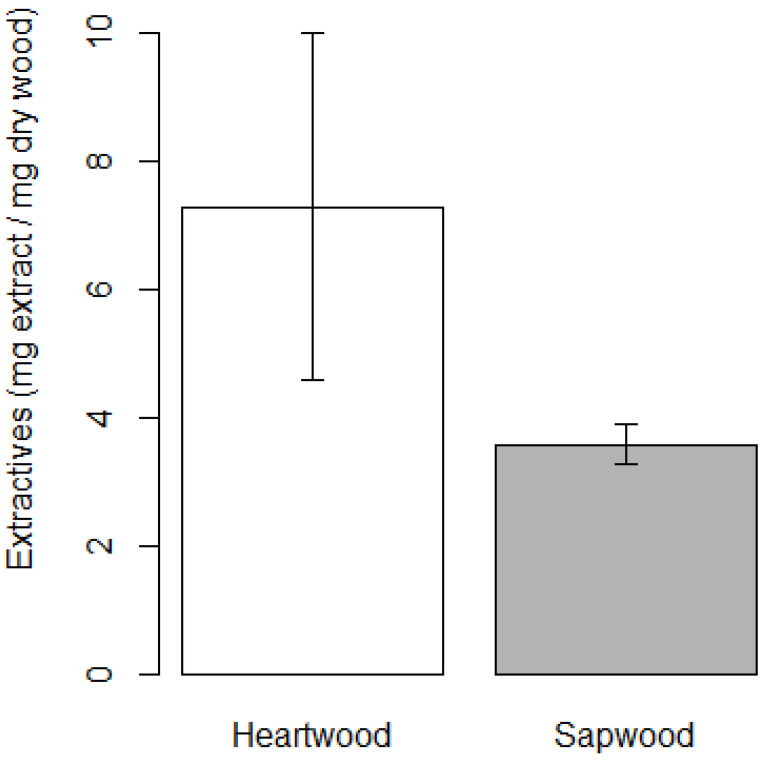
Soxhlet-extractable component content of Aomori hiba heartwood and sapwood. Data are expressed as mean ± SD (*n* = 3). Error bars are shown only as an approximate indicator of experimental variability.

Soxhlet extraction revealed a clear difference in extractable component content between heartwood and sapwood. As summarized in Figure 3, heartwood contained a higher amount of extractable components than sapwood. Although some variability was observed among heartwood samples, the mean extractable content of heartwood exceeded that of sapwood.

### 3.3. Relationship Between Fungal Susceptibility and Extractable Components

Comparison of biological and chemical results revealed a consistent trend. Heartwood, which exhibited stronger fungal susceptibility against *A. oryzae* (Figure 2), also contained a higher amount of Soxhlet-extractable components (Figure 3). Although specific active compounds were not identified in this study, the agreement between fungal susceptibility and extractable component content suggests that differences in extractives contribute to the distinct antifungal properties of heartwood and sapwood.

## 4. Discussion

### 4.1. Quantitative Image Analysis of Fungal Growth Inhibition

In this study, fungal growth inhibition against Aspergillus oryzae was quantitatively evaluated using grayscale-based digital image analysis of agar diffusion assays. As shown in Figure 2, the spatial distribution of fungal inhibition zones was converted into grayscale intensity profiles, enabling objective and reproducible quantification of inhibition behavior that cannot be adequately captured by visual inspection alone.

In particular, the intensity profiles revealed clear differences in both the extent and magnitude of fungal growth suppression between heartwood- and sapwood-derived samples. The gradual decrease in grayscale intensity toward the wood sample boundary reflects localized inhibition of fungal growth, providing spatially resolved information that is difficult to extract using conventional end-point or point-based measurements.

Importantly, Figure 2b demonstrates that the inhibition patterns differ not only qualitatively but also quantitatively, highlighting the value of image-derived metrics for comparing antifungal performance among wood samples. These results establish digital image analysis as a suitable tool for quantifying fungal growth inhibition in agar-based assays.

### 4.2. Relationship Between Image-Derived Metrics and Fungal Inhibition Behavior

The quantitative image analysis results provide direct insight into fungal inhibition behavior induced by wood extracts. The grayscale intensity profiles obtained in Figure 2 correspond to reduced fungal density in regions adjacent to the wood samples, indicating effective suppression of mycelial growth.

Unlike simple zone-diameter measurements, the present approach captures continuous spatial information, including gradual intensity transitions at inhibition boundaries. This enables more nuanced evaluation of antifungal effects, particularly in cases where inhibition zones are diffuse or irregular in shape.

Such image-based quantification is especially advantageous for fungal assays, where growth morphology and density variations often complicate manual evaluation. The present results demonstrate that grayscale-based analysis provides a robust and observer-independent means of linking visual fungal inhibition patterns to quantitative metrics.

### 4.3. Comparison with Previous Image-Based Microbiological Assays

Digital image analysis has been widely applied in microbiological research to quantify antimicrobial activity and microbial growth. Automated image-based screening methods have been reported for antibacterial assays, allowing objective evaluation of inhibition effects with improved reproducibility and throughput compared to manual inspection [21]. Similarly, fluorescence microscopy combined with digital image analysis has been established as a reliable approach for quantifying microorganisms, particularly when conventional culture-based counting is impractical [23].

The present study extends these image-based methodologies to fungal inhibition assays involving wood-derived materials. While previous studies have focused primarily on bacterial systems or fluorescence-based detection, the current work demonstrates that grayscale-based image analysis of standard agar diffusion assays can effectively quantify fungal growth inhibition without specialized labeling or instrumentation.

Thus, the proposed approach bridges established digital image analysis techniques in microbiology with wood science applications, providing a simple yet quantitative framework for evaluating antifungal activity.

### 4.4. Integration of Image Analysis with Extractive Content and Wood Tissue Differences

Differences in fungal susceptibility between heartwood and sapwood are generally attributed to variations in the concentration and composition of bioactive extractives. Numerous studies have reported that solvent-extractable compounds in wood tissues play a critical role in antimicrobial defense mechanisms [24].

Consistent with this established knowledge, the present study observed stronger fungal growth inhibition for heartwood samples, which are known to contain higher levels of antimicrobial extractives. However, rather than reiterating this qualitative distinction, the present work emphasizes the quantitative linkage between extractive-induced inhibition and image-derived metrics of fungal growth.

By integrating Soxhlet-extractable component measurements with quantitative image analysis, the study demonstrates how extractive content differences are reflected in spatially resolved inhibition patterns. This integration moves beyond conventional qualitative comparisons and provides a measurable connection between chemical extractives and biological response.

### 4.5. Scope, Limitations, and Applicability of the Present Study

Several limitations of the present study should be acknowledged. First, the chemical characterization of wood extractives was limited to bulk indicators, and individual antimicrobial compounds were not identified. As noted by the reviewer, a single chemical indicator cannot fully represent the compositional complexity of wood extractives. Accordingly, the scope of this work is positioned as a comparative, screening-level investigation rather than a comprehensive chemical analysis.

Second, the number of replicates was relatively small, and variability estimates should therefore be interpreted with caution. Third, image analysis was performed using a limited set of grayscale-based parameters.

Despite these limitations, the present study provides a proof-of-concept demonstration that quantitative digital image analysis can be effectively integrated with fungal susceptibility assays and extractive measurements to evaluate antifungal potential. From a sensing perspective, the proposed approach offers a practical balance between simplicity, reproducibility, and quantitative assessment, making it suitable for preliminary screening applications. Future studies incorporating multi-component chemical analyses, expanded datasets, and advanced image features will further strengthen the methodology.

## 5. Conclusions

Aomori hiba heartwood exhibits stronger fungal susceptibility against *Aspergillus oryzae* than sapwood. This difference is consistent with a significantly higher content of Soxhlet-extractable components in heartwood. The findings provide a clear and quantitative basis for distinguishing functional properties of heartwood and sapwood and support the effective utilization of Aomori hiba as a natural antimicrobial resource.

From a sensing perspective, the present study demonstrates that quantitative image analysis of fungal growth inhibition can function as a simple and effective image-based sensing method. By converting biologically induced inhibition zones into observer-independent numerical parameters, this approach enables reliable comparison of material-dependent antifungal properties. The consistency between image-based sensing outputs and Soxhlet-extractable component content indicates that the proposed method captures chemically relevant differences without requiring complex instrumentation. This image-based sensing framework may serve as a practical screening tool for evaluating mold susceptibility of wood-derived materials in food-related and hygienic contexts, particularly where rapid and low-cost assessment is required.

## Data Availability

Data are contained within the article.
